# TiF_4_ varnish protects the retention of brackets to enamel after *in vitro* mild erosive challenge

**DOI:** 10.1590/1678-7757-2017-0222

**Published:** 2018-05-03

**Authors:** Maria Isabel Dantas de Medeiros, Hugo Lemes Carlo, Rogério Lacerda dos Santos, Frederico Barbosa Sousa, Ricardo Dias de Castro, Renata Cristina Sobreira França, Fabíola Galbiatti de Carvalho

**Affiliations:** 1 Universidade Federal da Paraíba, Programa de Pós-Graduação em Odontologia, João Pessoa, Paraíba, Brasil; 2 Universidade Federal de Juiz de Fora, Departamento de Odontologia, Governador Valadares, Minas Gerais, Brasil

**Keywords:** Brackets, Fluorides, Shear bond strength, Tooth erosion

## Abstract

The effect of fluoride agents on the retention of orthodontic brackets to enamel under erosive challenge is little investigated.

**Objective:**

The aim of this study was to evaluate the effect of titanium tetrafluoride (TiF_4_) and sodium fluoride (NaF) agents on the shear bond strength of brackets to enamel and on the enamel microhardness around brackets under erosive challenge.

**Methods:**

Brackets were bonded to bovine incisors. Five groups were formed according to fluoride application (n=10): TiF_4_ varnish, TiF_4_ solution, NaF varnish, NaF solution and control (without application). The specimens were submitted to erosive challenge (90 s cola drink/2h artificial saliva, 4x *per* day for 7 days). Solutions were applied before each erosive cycle and varnishes were applied once. Vickers Microhardness (VHN) was obtained before and after all cycles of erosion and the percentage of microhardness loss was calculated. Shear bond strength, adhesive remnant index and polarized light microscopy were conducted after erosion. The data were analyzed by ANOVA, Tukey, Kruskal-Wallis and Mann-Whitney U tests (α=0.05).

**Results:**

The %VHN had no statistically significant differences among the experimental groups. However, considering the comparisons of all groups with the control group, TiF_4_ varnish showed the highest protection from enamel demineralization (effect size of 2.94, while the effect size for the other groups was >2.4). The TiF_4_ varnish group had significantly higher shear bond strength compared to other groups. There was no difference among groups for adhesive remnant index. Polarized light microscopy showed higher demineralization depth for the control group.

**Conclusions:**

Application of NaF and TiF_4_ agents during mild erosive challenge minimized the enamel mineral loss around brackets, however only the experimental TiF_4_ varnish was able to prevent the reduction of shear bond strength of brackets to enamel.

## Introduction

Dental erosion is the teeth mineral loss due to a chemical process, by exogenous or endogenous acids, without bacterial involvement^18^. The prevalence of dental erosion among 12 to 21 years-old-students in the world population varies approximately between 15 to 75%, with mild erosion being the most prevalent condition^1^ ^,^ ^7^ ^,^ ^14^. Currently, the most important acid sources come from dietary habits due to the increased consumption of soft drinks by the population^18^.

Consumption of acidic beverages decreases the pH in the oral environment, and factors other than pH, such as type of acid, pKa, titratable acidity, buffering capacity and temperature of acidic beverages can also influence on its erosive potential, causing enamel demineralization around brackets and interfering in their retention to enamel^12^ ^,^ ^22^. Thus, the topical application of fluoride is also recommended to minimize the enamel demineralization and to improve the shear bond strength of brackets to enamel^12^.

Sodium fluoride (NaF) is a monovalent fluoride compound and the most commonly found fluoride salt in toothpastes, mouthwashes and varnishes^24^. The NaF has shown positive results in the reduction of enamel erosion progression^6^ ^,^ ^13^ and its protective effect is associated with the precipitation of calcium fluoride material on eroded dental surfaces, especially when used in high concentration and acidic formulation^13^ ^,^ ^18^. As the anti-erosive effect of conventional monovalent fluorides requires a very intensive fluoridation regime^17^, current studies have focused on polyvalent metal ions of fluoride compounds that might have higher efficacy, as in the case of titanium tetrafluoride (TiF_4_)^13^ ^,^ ^17^. Studies have demonstrated that TiF_4_ increases the uptake of fluoride because of its acidic pH and can form a glaze-like surface layer that acts as an acid-resistance diffusion barrier^4^ ^,^ ^19^ ^,^ ^23^.

Although some studies have shown that the retention of orthodontic brackets to enamel is decreased when subjected to erosive challenge^12^ ^,^ ^22^, there have been no data published concerning whether NaF and TiF_4_ agents (varnish and solution) can protect the retention of metal orthodontic brackets to enamel during erosive challenge. Thus, the aim of this study was to evaluate the effect of TiF_4_ and NaF agents on the shear bond strength of brackets to enamel and on the enamel microhardness around brackets under erosive challenge. The hypotheses tested were that the TiF_4_ and NaF agents (varnish and solution) applied to enamel during an erosive challenge can minimize: 1) the demineralization of enamel around orthodontic brackets, as measured by a microhardness test and visualized by polarized light microscopy; 2) the decrease of the shear bond strength of metal orthodontic brackets on enamel, as measured by the shear bond strength test and the adhesive remnant index (ARI).

## Materials and methods

### Specimen preparation

Fifty freshly extracted lower bovine incisors were used in this study. The teeth were cleaned and the buccal surfaces were ground flat with SiC paper discs (400, 600 and 1200 grids) to expose the enamel bonding. The specimens were allocated into five groups (n=10) according to the fluoride application:

1. experimental TiF_4_ varnish group (TiF_4_ V) – FGM, Joinville, SC, Brazil;
2. experimental TiF_4_ solution group (TiF_4_ sol) – 4 g power TiF_4_ (Sigma-Aldrich, St. Louis, MO, USA) dissolved in 100 mL deionized water;
3. NaF varnish group (NaF V) – Duraphat - Colgate Palmolive Ltd., São Bernardo, SP, Brazil;
4. NaF solution group (NaF sol) – FlúorSol Clear – Dentsply, Petrópolis, RJ, Brazil;
5. Control group – without fluoride application.

**Figure 1:**
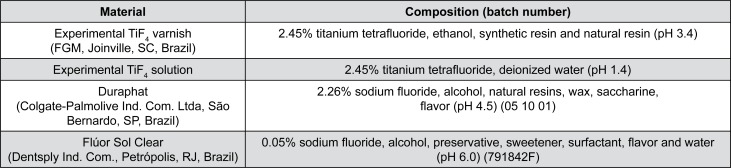
Compositions of fluoride agents tested in the study

The pH of solutions was measured by electrodes and the pH of varnishes were informed by the manufacturer. The composition of materials is described in Figure 1.

### Application of brackets

The roots were vertically embedded in acrylic resin (Vipi Flash, Pirassununga, SP, Brazil) up to the clinical crown level, using a set-square supported on the buccal surface to maintain the enamel surface perpendicular to the base of the acrylic resin. After prophylaxis with pumice and water, the specimens were etched with 35% phosphoric acid gel for 30 s, washed and dried by air-blowing. The primer and the resin of Transbond™ XT Light cured system (3M Unitek, Monrovia, CA, USA) were used to bond the maxillary central incisor bracket (Edgewise system, Morelli, Sorocaba, SP, Brazil) in the central area of the middle third of the buccal surface. The resin excess was removed. Light curing was performed for 60 s by LED (1200 mW/cm^2^ – Radii Cal. SDI, Bayswater, Victoria, Australia).

### Fluoride treatment

The enamel was covered with acid-resistant nail varnish (Colorama, CEIL Ltda., SP, Brazil) around the bracket at a distance of 2 mm. This area was submitted to application of fluoride agents and erosive challenge. The specimens were immersed in artificial saliva for 24 h^16^, the saliva was produced according to study held by McKnight-Hanes and Whitford^20^ (1992). Subsequently, the fluoride agent was applied on the enamel surface around the bracket using a 0.3 mL insulin syringe (BD Ultra-fine, Franklin Lakes, NJ, USA) to standardize the amount applied.

For groups 1 and 3, 20 μL of each fluoride varnish was applied on enamel around the bracket and spread with a microbrush. Samples were immersed in artificial saliva for 6 h to simulate clinically the contact time with the tooth surface^19^. Afterwards, the varnishes were carefully removed using a scalpel blade. For groups 2 and 4, 20 μL of each solution was applied on enamel around the bracket for 1 min. In the control group, no product was applied. The varnishes were applied only once before the erosive challenge, and solutions were applied before each erosive cycle^19^ ^.^

### Mild erosive challenge

Specimens were immersed in Coca-Cola (Coca-Cola, SP, Brazil – pH 2.3), using separate containers (30 mL/specimen) at room temperature, 4 times *per* day for 90 s each time^16^. After thorough rinsing with deionized water, specimens were immersed in artificial saliva, pH 7.0 (30 mL/specimen), at room temperature for 2 h, between erosive challenges and overnight. This erosive challenge was repeated for 7 days and the specimens were stored in 100% humidity for shear bond strength and microhardness tests.

### Shear bond strength test (SBS)

The direction of the debonding force was parallel to the enamel surface in an occlusogingival direction. A stainless steel rod with a chisel configuration was used for bracket debonding in a universal testing machine (Instron model 4411; Canton, MA, USA). Crosshead speed was 0.5 mm/min. The SBS was described in MPa.

### Microhardness test

A microdurometer (HMV II; Shimadzu Corporation, Kyoto, Japan) was used with a Vickers indentator, under a 1.961 N load for 15 s. The area selected for indentation was the enamel located in the direction of the bracket slot, at a distance of 50 μm from the area of bracket debonding. The enamel had five indentations made in the mesial and distal regions of the bracket in the area described. Each indentation was at a distance of 100 μm from the other. The mean of the vickers microhardness values (VHN) was obtained. Measurements were conducted before and after erosive challenge in the same location. In addition, the percentage of vickers microhardness loss (%VHN) was calculated using the following formula^5^.

%VHN=100(VHN_F_–VHN_I_/VHN_I_),

where VHN_I_ is the average of the initial (baseline) microhardness measurements, and VHN_F_ is the average of the final (after erosive challenge) microhardness values.

### Evaluation of adhesive remnant index (ARI)

After removal of the brackets, the ARI was observed using a stereomicroscope with 10x magnification by a single examiner (intra-examiner kappa=0.92) according to Artun and Bergland^2^ (1984): score 0) no composite left on the tooth; score 1) less than half of the composite left on the tooth; score 2) more than half of the composite left on the tooth; score 3) all composite left on the tooth, with distinct impression of the bracket mesh.

### Polarized light microscopy (PLM)

Teeth sections of 500 μm, containing an area of bracket adhesion and enamel, were obtained from each specimen. The sections were manually polished with SiC paper discs (600 and 1200 grits) under water refrigeration to a thickness of 100-120 μm. Polished tooth sections were placed in water and visualized under PLM (Axioskop 40, Carl Zeiss, Germany), and standard 35 mm photomicrographs were taken with 10x magnification.

### Sample size calculation

Based on a pilot test, a difference was predicted in the shear bond strength between the two groups with the highest difference of 4.0 MPa ± 3.0 MPa, corresponding to a Cohen's d effect size of 1.33. For the microhardness testing, a difference was predicted between groups with the highest difference of 33.5 VHN ± 25.0 VHN, corresponding to a Cohen's d effect size of 1.32. The Cohen's effect size d between the two groups with the highest difference can be used to calculate the sample size per group for a study with various groups using ANOVA^8^. Considering a two-tailed type I error of 5% (z score of 1.96), and statistical power of 80% (z score of 0.842), the calculated sample size using equation 12.2.1 of Cohen^8^ was 10 teeth *per* group for both shear bond strength and microhardness experiments.

### Statistical analysis

Data analysis was performed with the GraphPad Instat version 2.0 (GraphPad software, CA, USA) and Excel Microsoft software at a significance level of α=0.05. Two hypotheses of difference were tested: one related to %VHN, and other related to SBS. Because the variables tested satisfied the assumptions of equality and normal distribution (Bartlett and Kolmogorov–Smirnov tests, respectively), one-way ANOVA and Tukey's tests with corresponding Cohens' effect size d and its 95% confidence intervals (CI) were carried out for statistical comparisons of SBS and %VHN. The correlation between the group ranks and the size of the effect of %VHN (determined in relation to the effect size of %VHN between each group and the control group) was tested using the Spearman's rank correlation coefficient. Evaluation of ARI scores was carried out using the Kruskal-Wallis and Mann-Whitney *U* tests.

## Results

Following the recently published guidelines of the American Statistical Association^25^, our statistical analysis was not restricted to *p-*values. According to ASA guidelines^25^: (i), scientific conclusions should not be based whether a *p-*value passes as specific threshold; (ii) researchers should disclose the number of hypotheses explored during the study; and (iii) a *p-*value does not measure the size of an effect. Thus, the hypotheses tested in the statistical analysis of this study were reported using *p*-values, effect size (and its 95% CI), and power. The effect size measures the intensity of the difference (or correlation) between groups^8^.

**Figure 2:**
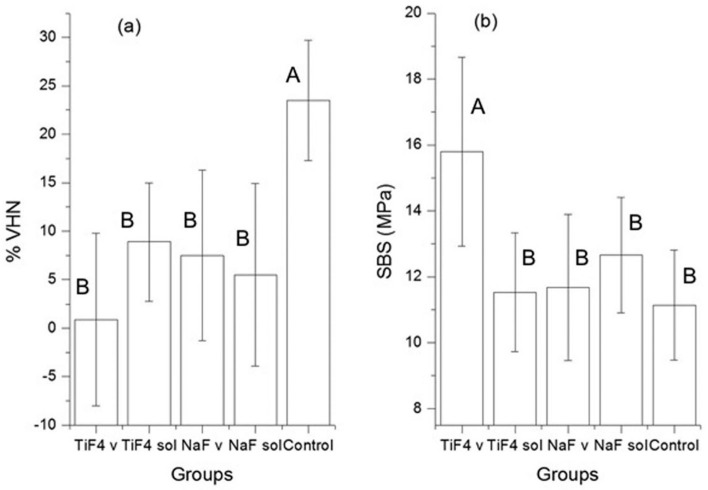
(a) Value plots (mean±SD) of microhardness percent loss (%VHN) for all groups. (b) Value plots of Shear Bond Strength (SBS) (mean±SD) in MPa. Different capital letters indicate statistical difference among groups (one-way ANOVA and Tukey's tests, p<0.05)

The mean values (± standard deviation) of SBS and %VHN are shown in Figure 2. The treatments affected significantly both SBS (*p*<0.0001, ANOVA) and %VHN (*p*=0.0002, ANOVA). For both SBS and %VHN, the effect size of the erosive challenge in the control group was the highest and in the TiF_4_ varnish group was the lowest. The pairwise comparisons are shown in Table 1. For all treatment groups, the %VHN was significantly lower (with a large effect size) compared with the control group. For other comparisons, no statistically significant differences were found, and the 95% CI either crossed the null hypothesis value or were very close to it (Table 1).

**Table 1:** Results of pairwise comparisons of microhardness percent loss (%VHN): Cohen's d effect size (ES) and its 95% confidence interval (upper limit of confidence interval/lower limit of confidence interval), and p-value (Tukey)

	TiF_4_ varnish	TiF_4_ solution	NaF varnish	NaF solution
TiF_4_ varnish	–––	–––	–––	–––
TiF_4_ solution	ES=1.05(1.98/0.11) p=0.0553	–––	–––	–––
NaF varnish	ES=0.75(1.65/-0.16) p=0.6653	ES=-0.19(0.69/-1.06) p=0.597	–––	–––
NaF solution	ES=0.50(1.39/-0.39) p=0.232	ES=0.43(1.32/-0.45) p=0.9588	ES=0.22(1.10/-0.66) p=0.9381	–––
Control	ES=2.94 (4.21/1.68) p<0.0000	ES=2.37(3.52/1.23) p<0.0004	ES=2.11(3.20/1.01) p<0.0000	ES=2.26(3.38/1.14) p<0.0000

Considering the different sizes of the positive effect of each treatment relative to the control group, the hypothesis that there was correlation between effect size (scalar data) and treatment type (ranked data) was tested. For that, the group ranks were: TiF_4_ varnish, rank=5; TiF_4_ solution, rank=4; NaF solution, rank=3; NaF varnish, rank=2, and control group, rank=1. The spearman's correlation of 0.99 indicates that the higher the group rank, the higher the size of the protection against enamel demineralization. The group ranks are positively correlated with the size of the protection against enamel demineralization. Thus, based on the Cohen's effect size d values, pairwise comparisons were ranked in the following order (from highest to lowest effect size): TiF_4_ varnish x control (effect size=2.94); TiF_4_ solution x control (effect size=2.37); NaF solution x control (effect size=2.26); NaF varnish x control (effect size=2.11), and control x control (effect size=0.0). The corresponding correlation coefficient was 0.99 (95% CI=0.999/0.903; *p*=0.0012; power=96.6%).

Regarding SBS, TiF_4_ varnish group had significantly higher SBS values (with large effect sizes) in all pairwise comparisons, while the other comparison showed no statistically significant differences (Table 2). The largest difference was between TiF_4_ varnish group and the control group.

**Table 2:** Results of pairwise comparisons of shear bond strength test: Cohen's d effect size (ES) and its 95% confidence interval (upper limit of confidence interval/lower limit of confidence interval), and p-value (Tukey)

	TiF_4_ varnish	TiF_4_ solution	NaF varnish	NaF solution
TiF_4_ varnish	–––	–––	–––	–––
TiF_4_ solution	ES=1.79(2.83/0.75) p=0.0004	–––	–––	–––
NaF varnish	ES=1.61(2.62/0.60) p=0.0007	ES=0.07(0.95/-0.80) p=0.9998	–––	–––
NaF solution	ES=1.33(2.30/0.34) p=0.0406	ES=0.64(1.53/-0.26) p=0.5042	ES=0.49(1.38/-0.40) p=0.6086	–––
Control	ES=1.99(3.07/0.92) p<0.003	ES=0.23(1.11/-0.65) p=0.9662	ES=0.28(1.16/-0.60) p=0.9887	ES=0.89(1.81/-0.03) p=0.8737

**Figure 3:**
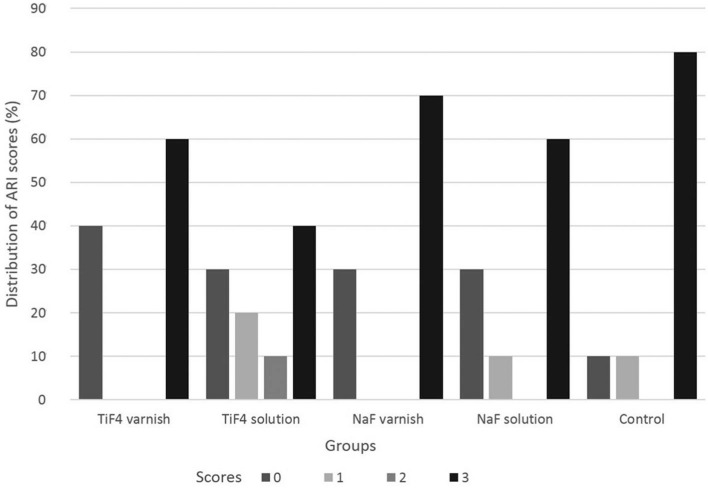
Distribution of Adhesive Remnant Index (ARI) scores (%) for each group

Figure 3 shows the percentage of ARI scores for each group. No significant difference was found among groups. All groups had large amounts of resin left on the tooth, with a distinct impression of the bracket mesh (score 3) (*p*=0.58).

**Figure 4:**
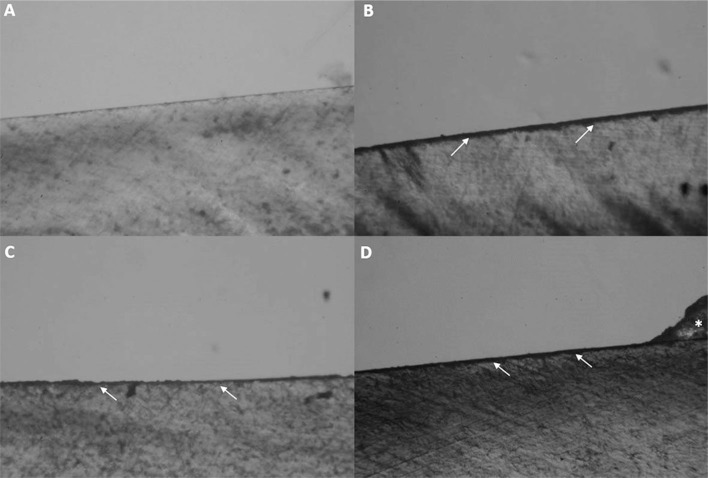
Polarized Light Microscopy images (10x). (A) Sound enamel. (B) Enamel demineralization in control; (C) NaF varnish; (D) and NaF solution groups. (→) Enamel demineralization. (*) Remaining resin left on enamel after shear bonding test

**Figure 5:**
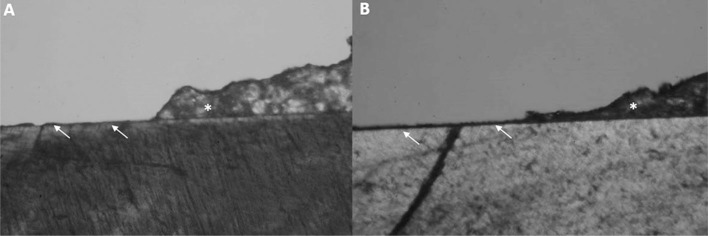
Polarized Light Microscopy images (10x). (A) Enamel demineralization in TiF_4_ varnish; (B) and TiF_4_ solution groups. (→) Enamel demineralization. (*) Remaining resin left on enamel after shear bond test

Figures 4 and 5 show PLM images of patterns of demineralization around brackets after fluoride application and erosive challenge. All groups showed enamel demineralization compared to sound enamel (Figure 4A). Control group (Figure 4B) showed a higher demineralization depth compared to other groups. NaF and TiF_4_ varnishes (Figures 4C and 5A) and the solutions (Figures 4D and 5B) groups showed similar demineralization patterns on the enamel.

## Discussion

The orthodontic bracket acts as an additional retention site for acidic substances, which contribute to tooth demineralization^10^ ^,^ ^15^ and decrease the bracket retention to the enamel surface^12^ ^,^ ^22^. This study was the first to evaluate solutions and varnishes containing monovalent or polyvalent metal ions fluoride compounds applied on enamel during erosive challenge and their influence on the retention of brackets to enamel and on the enamel microhardness around brackets.

In the oral cavity, the contact of the enamel with an acidic beverage is usually limited to a few seconds before clearance by saliva^3^. Thus, the dynamic erosive model with immersion in saliva simulated the typical consumption of soft drinks by individuals considered to be at risk for dental erosion^9^ ^,^ ^16^. Coca-Cola was chosen because it is widely consumed by the world population and has high erosive potential due to its low pH^12^ ^,^ ^21^ ^,^ ^22^. The immersion times of specimens in acidic beverages vary widely among studies. Oncag, Tuncer and Tosun^22^ (2005) and Navarro, et al.^21^ (2011) used longer times of immersion in soft drinks. However, the immersion time used in these studies^21^ ^,^ ^22^ does not represent the clinical situation, and they did not test the effect of fluoride agents. In general, studies that evaluated the anti-erosive effect of fluoride agents on enamel performed the fluoride application during 4-5 days of dynamic erosive challenge with immersion in acid beverage from 90 s to 5 min^9^ ^,^ ^16^ ^,^ ^19^.

This study found that after fluoride application and mild erosive challenge, the treatments had significant effect on the reduction of VHN values. While all treatment groups had significantly lower reduction in VHN values compared to the control group, they did not differ from each other (Table 1). However, the high correlation between effect size and treatment type (Spearman's correlation results) suggests that the protection against erosive challenge is expected to be the highest with TiF_4_ varnish group. This is consistent with other studies, which also showed better results with the TiF_4_ varnish compared to the NaF varnish, NaF solution and TiF_4_ solution to protect the enamel against erosion^16^ ^,^ ^19^. The fluoride concentration (2.45%) and pH (3.4) of TiF_4_ varnish (Figure 1) may have influenced the VHN results, because high concentration and low pH could increase the fluoride uptake by enamel^13^. However, the reduction in enamel erosion by TiF_4_ agents is primarily attributed to the precipitation of a metal-rich layer on a tooth surface^16^ ^,^ ^19^.

Titanium ions may substitute calcium in the apatite lattice and show a strong tendency to complex with phosphate groups, forming a new compound (hydrated hydrogen titanium phosphate) or organometallic complexes^4^ ^,^ ^23^. This reaction forms a glaze-like surface layer that can act as an acid-resistance diffusion barrier^4^ ^,^ ^23^. The different surface effects between TiF_4_ formulations can be related to the better ability of the varnish to adhere on enamel compared to solution; therefore, the varnish was applied only once whereas the solution was frequent^19^. This adherence characteristic of varnish prolongs the reaction between TiF_4_ and enamel^19^.

A previous study showed that the experimental TiF_4_ varnish had better protective effect against erosion of enamel than the experimental NaF varnish with the same fluoride concentration (2.45%)^16^. Thus, our study chose to compare the protective effect of TiF_4_ varnish with a commercial varnish (Duraphat – NaF varnish 2.26%). However, the higher fluoride concentration of TiF_4_ varnish can be influenced in the results. The NaF varnish group was ranked lower than the TiF_4_ varnish group. Concerning the Cohen's effect size values of the protection in enamel against erosion, the difference between the TiF_4_ varnish and the NaF varnish group was the largest among treatments, being consistent with the aforementioned report. The difference can also be associated with the different protective layers formed on enamel. The calcium fluoride protective layer has been speculated to be less resistant to erosive challenge than the glaze-like layer^9^ ^,^ ^16^ ^,^ ^19^ ^,^ ^23^.

The lower %VHN of all groups compared to the control group (Figure 2a) showed that after erosion, the application of NaF or TiF_4_ agents (varnish and solution) minimized the enamel mineral loss around brackets, but they were not able to arrest mineral loss completely. These results were also demonstrated by PLM images. The control group seemed to have a demineralization depth higher than other groups (Figure 4B), and there was enamel demineralization after fluoride application, but with no apparent difference among NaF and TiF_4_ groups (Figures 4 C, D and 5 A, B).

The values of shear bond strength after erosion (Figure 2b) were higher than the values showed in other studies^21^ ^,^ ^22^, most likely due to the use of fluoride agents and the inferior immersion time of specimens in acidic beverage. The ARI results showed that the score of 3 was the most observed for all groups (Figure 3), indicating that the erosive challenge did not impair the bonding of resin to enamel and that the difference found among groups for shear bond strength can be associated to the fluoride agent used in each group. Thus, the TiF_4_ varnish group was the only one that showed statistically higher shear bonding strength of brackets to enamel after the erosive challenge compared to all groups (Figure 2b and Table 2). The glaze-like surface layer formed probably protected the demineralization of enamel, as explained before, and was able to prevent the decrease of shear bond strength caused by erosion. Fidalgo, et al.^11^ (2012) found that NaF fluoride treatments improved the shear bond strength of brackets to enamel after cariogenic challenge, because NaF forms fluoride hydroxyapatite, which is more resistant than hydroxyapatite^11^. However, our study showed that most likely the reaction of titanium ions with enamel apatite caused more protection from shear forces than the NaF reaction.

Formulations with low concentration of fluoride, as in toothpastes, had minimal or no anti-erosive effect^13^ ^,^ ^18^. Although fluoride varnish requires a professional application and the cost-effectiveness can be higher than home care products, the topical fluoride varnish treatments have a surface and a sub-surface effect, which may be relevant in the prevention of dental erosion^24^. Furthermore, fluoride varnishes are easy to apply, safe and well-tolerated by infants and children^16^. The hypotheses tested were partially accepted because the application of NaF and TiF_4_ agents (varnish and solution) during erosive challenge minimized the enamel mineral loss around brackets, but only TiF_4_ varnish was able to protect the shear bond strength of brackets to enamel. Although this study has been conducted *in vitro*, the experimental TiF_4_ varnish seemed to be a promising agent to reduce enamel loss and to improve the retention of brackets to enamel under mild erosive conditions. However, *in vivo* studies should be conducted to verify the efficacy of TiF_4_ varnish in preventing enamel demineralization and retention of brackets to enamel during comprehensive orthodontic treatment of patients with dental erosion diagnosis.

## Conclusions

Application of NaF and TiF_4_ agents (varnish and solution) during mild erosive challenge minimized the enamel mineral loss around brackets, however only experimental TiF_4_ varnish was able to prevent the reduction of shear bond strength of brackets to enamel.

## References

[1] Alves LS, Brusius CD, Damé-Teixeira N, Maltz M, Susin C (2015). Dental erosion among 12-year-old schoolchildren: a population-based cross-sectional study in South Brazil. Int Dent J.

[2] Artun J, Bergland S (1984). Clinical trials with crystal growth conditioning as an alternative to acid-etch enamel pretreatment. Am J Orthod.

[3] Attin T, Wegehaupt FJ (2014). Methods for assessment of dental erosion. Monogr Oral Sci.

[4] Büyükyilmaz T, Ogaard B, Rølla G (1997). The resistance of titanium tetrafluoride-treated human enamel to strong hydrochloric acid. Eur J Oral Sci.

[5] Carvalho FG, Vieira BR, Santos RL, Carlo HL, Lopes PQ, Lima BA (2014). *In vitro* effects of nano-hydroxyapatite paste on initial enamel carious lesions. Pediatr Dent.

[6] Carvalho AC, Sanches RP, Martin AA, Espírito Santo AM, Soares LE (2011). Energy dispersive X-ray spectrometry study of the protective effects of fluoride varnish and gel on enamel erosion. Microsc Res Tech.

[7] Chu CH, Ng A, Chau AM, Lo EC (2015). Dental erosion and caries status of chinese university students. Oral Health Prev Dent.

[8] Cohen J (1988). Statistical power analysis for the behavioral sciences.

[9] Comar LP, Cardoso CB, Charone S, Grizzo LT, Buzalaf MA, Magalhães AC (2015). TiF4 and NaF varnishes as anti-erosive agents on enamel and dentin erosion progression *in vitro*. J Appl Oral Sci.

[10] Damon PL, Bishara SE, Olsen ME, Jakobsen JR (1996). Effects of fluoride application on shear bond strength of orthodontic brackets. Angle Orthod.

[11] Fidalgo TK, Pithon MM, Santos RL, Alencar NA, Abrahão AC, Maia LC (2012). Influence of topical fluoride application on mechanical properties of orthodontic bonding materials under pH cycling. Angle Orthod.

[12] Hammad SM, Enan ET (2013). *In vivo* effects of two acidic soft drinks on shear bond strength of metal orthodontic brackets with and without resin infiltration treatment. Angle Orthod.

[13] Huysmans MC, Young A, Ganss C (2014). The role of fluoride in erosion therapy. Monogr Oral Sci.

[14] Isaksson H, Birkhed D, Wendt LK, Alm A, Nilsson M, Koch G (2014). Prevalence of dental erosion and association with lifestyle factors in Swedish 20-year olds. Acta Odontol Scand.

[15] Keçik D, Çehreli SB, Şar Ünver B (2008). Effect of acidulated phosphate fluoride and casein phosphopeptide-amorphous calcium phosphate application on shear bond strength of orthodontic brackets. Angle Orthod.

[16] Levy FM, Magalhães AC, Gomes MF, Comar LP, Rios D, Buzalaf MA (2012). The erosion and abrasion-inhibiting effect of TiF4 and NaF varnishes and solutions on enamel *in vitro*. Int J Paediatr Dent.

[17] Levy FM, Rios D, Buzalaf MA, Magalhães AC (2014). Efficacy of TiF4 and NaF varnish and solution: a randomized in situ study on enamel erosive-abrasive wear. Clin Oral Investig.

[18] Lussi A, Carvalho TS (2015). The future of fluorides and other protective agents in erosion prevention. Caries Res.

[19] Magalhães AC, Kato MT, Rios D, Wiegand A, Attin T, Buzalaf MA (2008). The effect of an experimental 4% TiF4 varnish compared to NaF varnishes and 4% TiF4 solution on dental erosion *in vitro*. Caries Res.

[20] McKnight-Hanes C, Whitford GM (1992). Fluoride release from three glass ionomer materials and the effects of varnishing with or without finishing. Caries Res.

[21] Navarro R, Vicente A, Ortiz AJ, Bravo LA (2011). The effects of two soft drinks on bond strength, bracket microleakage, and adhesive remnant on intact and sealed enamel. Eur J Orthod.

[22] Oncag G, Tuncer AV, Tosun YS (2005). Acidic soft drinks effects on the shear bond strength of orthodontic brackets and a scanning electron microscopy evaluation of the enamel. Angle Orthod.

[23] Ribeiro CC, Gibson I, Barbosa MA (2006). The uptake of titanium ions by hydroxyapatite particles-structural changes and possible mechanisms. Biomaterials.

[24] Sar Sancakli H, Austin RS, Al-Saqabi F, Moazzez R, Bartlett D (2015). The influence of varnish and high fluoride on erosion and abrasion in a laboratory investigation. Aust Dent J.

[25] Wasserstein RL, Lazar NA (2016). The ASA's statement on p-values: context, process, and purpose. Am Stat.

